# Addressing Gaps in the Chicago Classification Version 4.0: Defining an Optimal Intrabolus Pressure Measurement on Esophageal Manometry

**DOI:** 10.1111/nmo.70320

**Published:** 2026-04-12

**Authors:** Muhammed M. Alikhan, Aidan D. Smires, John E. Pandolfino, Wenjun Kou, Jacob M. Schauer, Neelesh A. Patankar, Dustin A. Carlson

**Affiliations:** ^1^ Kenneth C. Griffin Esophageal Center of Northwestern Medicine, Department of Medicine, Division of Gastroenterology and Hepatology Feinberg School of Medicine, Northwestern University Chicago Illinois USA; ^2^ Division of Biostatistics, Department of Preventive Medicine Feinberg School of Medicine, Northwestern University Chicago Illinois USA; ^3^ Department of Mechanical Engineering, McCormick School of Engineering Northwestern University Evanston Illinois USA

**Keywords:** dysphagia, esophagus, manometry, motility, impedance, peristalsis

## Abstract

**Background:**

Intrabolus pressure (IBP) reflects the pressure within the esophageal lumen during bolus transit and serves as a physiologic marker of outflow resistance at the esophagogastric junction (EGJ). The lack of a standardized or validated method to measure IBP is a critical limitation for interpreting high resolution manometry (HRM) and identification of clinically relevant EGJ outflow obstruction (EGJOO).

**Methods:**

Three distinct cohorts, “Controls”, Normal motility”, and “conclusive EGJOO” were selected from a prospectively enrolled cohort of adult patients. All patients had at least undergone HRM with impedance (HRIM), and functional lumen impedance probe (FLIP) testing. 4D HRM analysis was performed blinded to clinical characteristics. 4D HRM IBP results were assessed on a per‐swallow and also on a per‐patient level. Receiver operating curve (ROCs) to assess each metrics prediction of conclusive EGJOO vs. not EGJOO (normal motility and controls) were utilized for the per‐swallow analysis.

**Key Results:**

33 controls, 35 normal motility, and 15 conclusive EGJOO patients were included. Swallow level analysis was conducted on 156 swallows, 165 swallows, and 61 swallows from each group, respectively. Per‐swallow analysis demonstrated differences between conclusive EGJOO, normal motility, and controls for all ten IBP measures (P‐values < 0.001), with greater IBP measures in conclusive EGJOO than in normal motility and controls. The 1 s max IBP had the greatest AUROC.

**Conclusions & Inferences:**

Standardized measurement of IBP using an optimized method (1‐s max IBP) within the 4D‐HRM framework with impedance‐confirmed bolus tracking and phase‐specific measures represents a physiologically grounded and clinically meaningful advance in HRIM interpretation.

## Key Points

- The current measurement of intrabolus pressure (IBP) on esophageal manometry (HRM) lacks standardization under the Chicago Classification v4.0 scheme.
- Standardized measurement of IBP is crucial for accurately identifying patients with EGJ outflow obstruction (EGJOO), and subsequent decisions on management.
- The maximal, non‐contiguous pressure measurement over 1 s was found to be the highest performing metric in measurement of IBP.

## Graphical abstract

This graphical abstract illustrates included patients in the study on the top panel, a graphical representation of intrabolus pressure (IBP) measurement techniques in the bottom left panel, and performance comparison by area under the receiver operating curve (AUROC) analysis of these various measurement techniques.

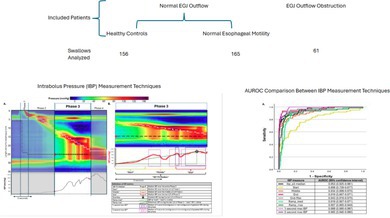

## 1 Introduction

Intrabolus pressure (IBP) reflects the pressure within the esophageal lumen during bolus transit and serves as a physiologic marker of outflow resistance at the esophagogastric junction (EGJ) [1, 2]. IBP is recognized as a valuable indicator of EGJ obstruction, including incorporating elevated IBP as a criterion for esophagogastric junction outflow obstruction (EGJOO) in the Chicago Classification version 4.0 (CCv4.0) [2, 3, 4, 5]. However, the lack of a standardized or validated method to measure IBP is a critical limitation for interpreting HRM and identification of clinically relevant EGJOO. Previous IBP measurement approaches using single‐sensor methods or HRM region‐based analyses assumed esophageal bolus presence during measures and automated software approaches can overlap with contractile wave, with associated concern regarding the accuracy of these measures [2, 5]. This can have clinical implications, especially if isobaric contour is set at an arbitrary value.

Advances in impedance‐integrated HRM provide an opportunity to improve measurement of IBP. We developed a technique termed 4D HRM that leverages nadir impedance, which was defined during the conception of automated impedance manometry (AIM), to confirm the presence and location of the bolus during each swallow [6]. This enabled calculation of an accurate IBP with the potential to improve diagnostic precision for EGJOO [7, 8]. While we previously described swallow phase‐specific IBP as a median IBP value over the entire duration of each swallow phase, we hypothesized that further refinement of the IBP measurement technique could enhance its diagnostic application. The aim of the present study was to define an optimal IBP measurement strategy using impedance‐confirmed bolus presence and to assess its ability to discriminate conclusive EGJOO from both normal motility and asymptomatic controls, thereby addressing a critical unmet need within the CCv4.0 diagnostic framework.

## 2 Methods

### 2.1 Subjects

Specific cohorts were selected to identify conclusive cases of normal EGJ outflow (“controls” and “normal motility”) or EGJ outflow obstruction. “Controls” were from a previously described cohort of healthy asymptomatic volunteers who completed HRIM and FLIP with endoscopy [8]. “Normal motility” and “conclusive EGJOO” were selected from a previously described prospectively enrolled cohort of adult patients with non‐obstructive (i.e., endoscopy‐negative) dysphagia that completed HRM with impedance (HRIM), timed barium esophagram (TBE), and functional lumen impedance probe (FLIP) testing. “Normal motility” was defined by normal HRM (per CCv4.0), normal FLIP (per Dallas consensus), and normal TBE (no column by 1 min and passage of a 12.5 mm barium tablet) [9, 10]. These patients were considered to have a functional etiology of their dysphagia. “Conclusive EGJOO” was defined by HRM (per CCv4.0; 4D HRM metrics were not previously applied for the HRM impression of EGJOO), abnormal retention on TBE and abnormal EGJ opening on FLIP, ie FLIP with reduced EGJ opening and TBE with at least > 8 cm column height at 1 min or tablet impaction, or FLIP with borderline EGJ opening and TBE with > 5 cm [3, 9, 10]. Informed consent was obtained and study protocols were approved by the Northwestern University Institutional Review Board.

### 2.2 HRIM Protocol and 4D HRM Analysis

**FIGURE 1:**
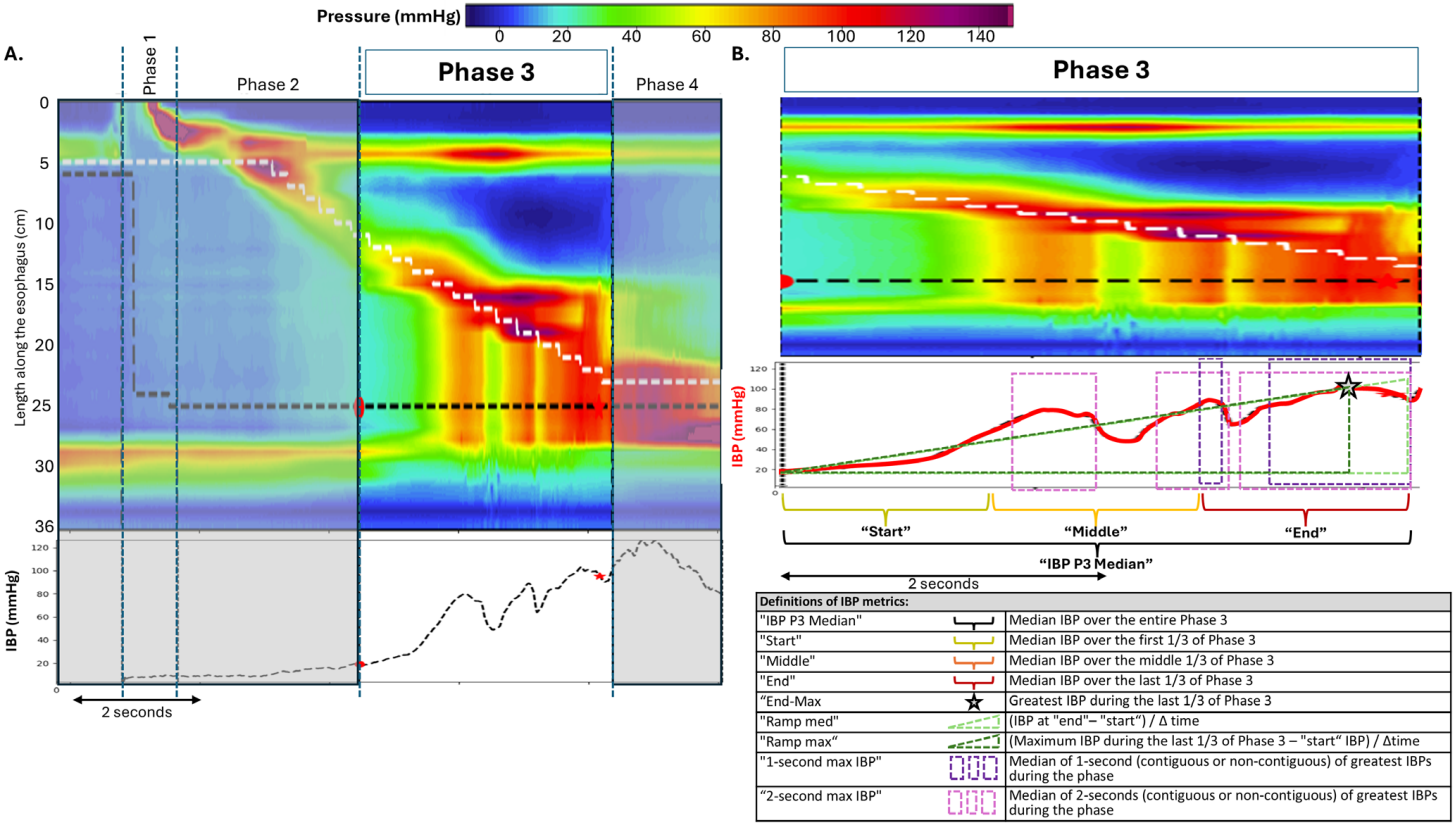
Intrabolus pressure (IBP) measures. A representative swallow (A) from a patient with conclusive EGJ outflow obstruction to demonstrate the four phases of bolus transit, with phases 1, 2, and 4 grayed out as the focus for IBP measures was in Phase 3 (peristalsis/esophageal emptying). Phase 3 (A,B) represents the period when the contractile wave is propagating from the transition zone (start of phase 3) to the contractile deceleration point (end of phase 3). The Intra‐bolus pressure (IBP) was measured at the nadir impedance black dashed lined overlying the topography plots to optimize determining sites of bolus presence. The specific IBP measures tested to determine an optimal method are depicted. (B) Ramp‐med and Ramp‐max overlap in this example swallow. IBP increases can be appreciated as the compartmentalized pressure on the topography between the contractile wave and the EGJ reflecting EGJ outflow obstruction in this example swallow. Figure used with permission from the Esophageal Center of Northwestern.

A Medtronic HRIM assembly with 36 circumferential pressure sensors at 1‐cm interval, and 18 impedance segments at 2‐cm intervals was utilized with studies performed per CCv4.0 protocol as previously described [3, 8]. 4D HRM analysis was performed blinded to clinical characteristics (Wenjun Kou, Muhammed M. Alikhan, Aidan D. Smires). Each HRIM study had the first 5 supine swallows with adequate impedance readings analyzed to assess IBP via various iterations (Figure 1) per the 4D HRM framework. As previously described, data from each swallow in Manoview software were exported into a customized analysis software with manual input of landmarks to identify four phases of esophageal bolus transport [7, 8, 11]. Only swallows with normal peristalsis (per CCv4.0; i.e., distal contractile integral > 450 mmHg•mm•s without large peristaltic break) were included for analysis [3]. The nadir impedance was tracked by the analysis program and was utilized to define bolus presence over the total course of the swallow. For IBP measurement, phase 3 (peristalsis/esophageal emptying) was the specific focus as this phase of peristalsis would be expected to yield elevated IBP in the setting of EGJOO, i.e., bolus pressurized between the contractile wave and EGJ outflow resistance; Figure 1. A safeguard to ensure IBP measures reflected bolus pressurization instead of intersecting the contractile wavefront was cessation of the IBP measurement 0.2 s prior to the contractile deceleration point (CDP; end of phase 3). The program output was also manually reviewed with adjustment of the IBP measurement window as needed to avoid time points of nadir impedance intersecting with the contractile pressure wave or the EGJ.

4D HRM IBP results were assessed on a per‐swallow (lack of data independence acknowledged) and also on a per‐patient level. Receiver operating curve (ROCs) to assess each metrics prediction of conclusive EGJOO vs. not EGJOO (normal motility and controls) were utilized for the per‐swallow analysis. Cohorts were compared using Kruskal‐Wallis/Mann–Whitney U. A two‐tailed *p* < 0.05 was considered to meet statistical significance, a Bonferroni correction was applied when necessary to address multiple comparisons.

## 3 Results

### 3.1 Subjects

33 controls (mean (SD) age 30 (6) years; 23 (70%) female), 35 normal motility (mean (SD) age 45 (17) years; 27 (77%) female), and 15 conclusive EGJOO (mean (SD) age 53 (15) years; 5 (33%) female) patients were included. Swallow level analysis was conducted on 156 swallows, 165 swallows, and 61 swallows from each group, respectively.

**FIGURE 2:**
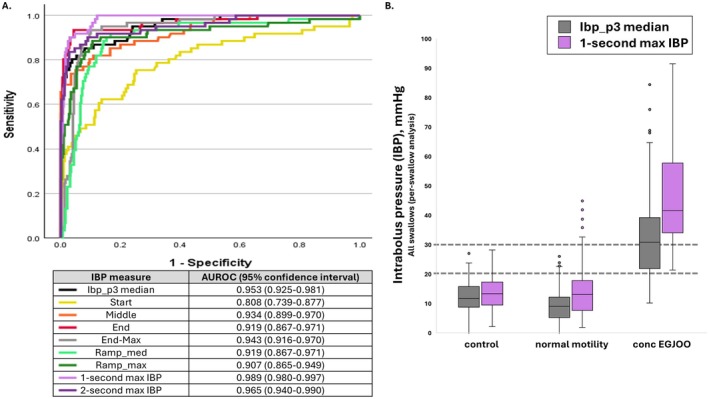
Assessment of Intrabolus pressure (IBP) measures. (A) Receiver operating curve demonstrating per‐swallow analysis for prediction of conclusive EGJ outflow obstruction vs. not‐EGJ outflow obstruction (i.e., healthy controls and normal motility). (B) Per‐swallow analysis of the IBP Phase 3 (p3) median and 1‐s max IBP between the three cohorts. “○” indicate outlier values.

Per‐swallow analysis demonstrated differences between conclusive EGJOO, normal motility, and controls for all ten IBP measures (*p* < 0.001), with greater IBP measures in conclusive EGJOO than in normal motility and controls; Figure 2. The 1 s max IBP had the greatest AUROC 0.989 (95% CI 0.980–0.997); Figure 2. Median (5‐95th percentiles) for the 1 s max IBP was 14 (4–25) mmHg for controls, 14 (4–32) mmHg for normal motility, and 46 (25–95) mmHg for conclusive EGJOO; Figure 2.

Per‐patient analysis demonstrated that when applying the 1 s max IBP, 27% of healthy controls had ≥ 20% swallows with IBP > 20 mmHg and zero (0%) had ≥ 20% swallows > 30 mmHg. 46% of patients in the normal motility cohort had ≥ 20% swallows with IBP > 20 mmHg and 11% had ≥ 20% swallows > 30 mmHg. 100% of patients in the conclusive EGJOO cohort had ≥ 20% swallows with IBP > 20 mmHg and 93% had ≥ 20% swallows > 30 mmHg.

## 4 Discussion

This proof‐of‐concept study defined an optimal method for measuring IBP using HRIM, leveraging the novel 4D HRM framework and testing on rigorously‐defined cohorts of symptomatic patients and asymptomatic controls. The 4D HRM framework enabled impedance‐confirmed bolus localization by tracking nadir impedance across the phases of esophageal bolus transit. Focusing on Phase 3 as bolus pressurization is expected to occur during peristalsis/emptying in the setting of a relevant EGJ obstruction, the best performing method to discriminate between conclusive EGJOO and normal EGJ outflow was the 1‐s maximum IBP. These findings directly address an important limitation of CCv4.0, in which IBP is included as a criterion for EGJOO, but without a standardized or validated measurement approach.

Accurate IBP assessment requires precise temporal and spatial localization of bolus pressurization. Prior methods carried limitations based on assumptions of bolus presence and in some cases confounded by measurement overlap with the peristaltic wavefront and EGJ [2, 5, 12]. The 4D HRM approach tracks bolus presence and is based on physiologic concepts of bolus transit, which facilitated sampling pressures that reflect increases in IBP associated with EGJ obstruction. While all of the 4D HRM IBP effectively differentiated normal from conclusive EGJOO, the 1‐s maximum IBP emerged as the optimal metric. This measure was calculated as the greatest (maximum) IBP that totaled 1 s in duration and could be contiguous or non‐contiguous, i.e., in a manner reminiscent of the integrated relaxation pressure (IRP) calculation. Supported by the results, this method is also conceptually appealing as it is less prone to artifact than single time‐point measure and yet also captures the most physiologically relevant period(s) of sustained bolus pressurization during esophageal emptying, rather than diluting peak effects through longer, less specific time windows.

Additionally, the 4D HRM analysis requires accurate identification of physiologic landmarks, though these are all familiar, included in the CCv4 analysis scheme [3]. Further, we intentionally avoided proposing distinct IBP thresholds within this method‐optimization study due to the relatively small cohort sizes. A goal of future study with larger patient cohorts will be to better determine clinically relevant thresholds for IBP, possibly incorporating probability from both an IBP threshold and number or percentage of swallows involved. However, even with the refined IBP approach, there remained overlap in IBP between EGJOO and non‐EGJOO, supporting ongoing study to improve approaches to inconclusive HRMs. However, this also supports a cautious approach when encountering isolated ’abnormalities’ that may not be clinically relevant within the course of a clinical evaluation including HRIM.

Key strengths of this work include the use of prospective research cohorts with well‐defined presence or absence of EGJ outflow obstruction (including applying measures that are independent of HRM), as well as the novel 4D HRM analytic approach. The analysis was intentionally limited to swallows with intact peristalsis, as adequate contractile vigor is necessary to generate intrabolus pressurization in the setting of increased EGJ resistance. While this limits direct generalizability to weak or failed swallows, it should be recognized that IBP measures may underestimate EGJ obstruction in this setting. However, this approach brings to light the concept that EGJOO should be considered in the context of peristaltic function and should be defined based on level of IBP. The classic high IBP EGJOO that is part of the CC 4.0 classification is associated with intact peristalsis and likely normal anatomy and mechanics. In contrast, an entity with low IBP related to ineffective or absent contractility and impaired EGJ opening, akin to type I achalasia, may be missed using thresholds of IBP alone to define obstruction. Thus, defining EGJOO may be helped by assessing bolus retention using impedance or esophagram to better codify fluid dynamics during swallowing.

In conclusion, standardized measurement of IBP using an optimized method (1‐s max IBP) within the 4D HRM framework with impedance‐confirmed bolus tracking and phase‐specific measures represents a physiologically grounded and clinically meaningful advance in HRIM interpretation. Further work is needed to incorporate a validation cohort utilizing this approach, and a larger study is underway. Nonetheless, a studied approach to measuring IBP begins to address a critical gap in HRM interpretation systems and provides a foundation for future studies aimed at refining diagnostic thresholds and improving evaluation of inconclusive HRMs.

## Author Contributions

Muhammed M. Alikhan and Aidan D. Smires contributed to drafting of the manuscript, data analysis, data interpretation, and approval of the final version. John E. Pandolfino contributed to study concept and design, obtaining funding, editing the manuscript critically, data interpretation, and approval of the final version. Wenjun Kou, Jacob M. Schauer, and Neelesh A. Patankar contributed to data analysis, data interpretation, and approval of the final version. Dustin A. Carlson contributed to study concept and design, obtaining funding, drafting of the manuscript, data analysis, data interpretation, and approval of the final version.

## Funding

This work was supported by R01 DK137775 (John E. Pandolfino and Dustin A. Carlson) from the Public Health service.

## Conflicts of Interest

John E. Pandolfino: Medtronic (Speaking, Consulting, Patent, License); Sandhill Scientific/Diversatek (Consulting, Grant); Torax (Speaking, Consulting); EndoGastric Solutions (speaking, consulting); Laborie (Consulting); Phathom Pharmaceuticals (Speaking, Consulting). Wenjun Kou: BMS (Consulting), Calyx (Consulting), AstraZeneca (Consulting), Sanofi (Consulting). Dustin A. Carlson: Medtronic (Speaking, Consulting, License); Diversatek (Consulting); Braintree (Consulting); Medpace (Consulting); Phathom Pharmaceuticals (Speaking; Consulting); Regeneron/Sanofi (Speaking, Consulting); Laborie (Consulting). Other authors declare no conflicts.

## Data Availability

The data that support the findings of this study are available from the corresponding author upon reasonable request and appropriate approvals.

## References

[1] B. T. Massey , W. J. Dodds , W. J. Hogan , et al., “Abnormal Esophageal Motility. An Analysis of Concurrent Radiographic and Manometric Findings,” Gastroenterology 101, no. 2 (1991): 344–354. 2065909

[2] A. J. Bredenoord , A. Babaei , D. Carlson , et al., “Esophagogastric Junction Outflow Obstruction,” Neurogastroenterology and Motility 33 (2021): e14193, 10.1111/nmo.14193. 34120375

[3] R. Yadlapati , P. J. Kahrilas , M. R. Fox , et al., “Esophageal Motility Disorders on High‐Resolution Manometry: Chicago Classification Version 4.0((c)),” Neurogastroenterology and Motility 33, no. 1 (2021): e14058, 10.1111/nmo.14058. 33373111 PMC8034247

[4] J. Ren , B. T. Massey , W. J. Dodds , et al., “Determinants of Intrabolus Pressure During Esophageal Peristaltic Bolus Transport,” American Journal of Physiology 264, no. 3 Pt 1 (1993): G407–G413, 10.1152/ajpgi.1993.264.3.G407. 8460696

[5] F. Quader , C. Reddy , A. Patel , et al., “Elevated Intrabolus Pressure Identifies Obstructive Processes When Integrated Relaxation Pressure Is Normal on Esophageal High‐Resolution Manometry,” American Journal of Physiology. Gastrointestinal and Liver Physiology 313, no. 1 (2017): G73–G79, 10.1152/ajpgi.00091.2017. 28408642 PMC5538833

[6] N. Rommel , L. Van Oudenhove , J. Tack , et al., “Automated Impedance Manometry Analysis as a Method to Assess Esophageal Function,” Neurogastroenterology and Motility 26, no. 5 (2014): 636–645, 10.1111/nmo.12308. 24447538

[7] W. Kou , D. A. Carlson , P. J. Kahrilas , et al., “Normative Values of Intra‐Bolus Pressure and Esophageal Compliance Based on 4D High‐Resolution Impedance Manometry,” Neurogastroenterology and Motility 34 (2022): e14423, 10.1111/nmo.14423. 35661346 PMC9529819

[8] E. Goudie , W. Kou , J. E. Pandolfino , et al., “Four‐Dimensional Impedance Manometry in Esophageal Motility Disorders,” American Journal of Gastroenterology 120, no. 5 (2025): 1019–1026, 10.14309/ajg.0000000000003151. 39422339 PMC12006448

[9] O. Z. Fass , J. E. Pandolfino , J. M. Schauer , et al., “Diagnostic Accuracy of Timed Barium Esophagram for Achalasia,” Gastroenterology 169 (2025): 63–72, 10.1053/j.gastro.2025.02.013. 40020937 PMC12185239

[10] D. A. Carlson , J. E. Pandolfino , R. Yadlapati , et al., “A Standardized Approach to Performing and Interpreting FLIP Panometry for Esophageal Motility Disorders: The Dallas Consensus,” Gastroenterology 168 (2025): 1114–1127.e5, 10.1053/j.gastro.2025.01.234. 39914779 PMC12104001

[11] W. Kou , D. A. Carlson , N. A. Patankar , P. J. Kahrilas , and J. E. Pandolfino , “Four‐Dimensional Impedance Manometry Derived From Esophageal High‐Resolution Impedance‐Manometry Studies: A Novel Analysis Paradigm,” Therapeutic Advances in Gastroenterology 13 (2020): 1756284820969050, 10.1177/1756284820969050. 33178334 PMC7592175

[12] J. M. Colizzo , S. B. Clayton , and J. E. Richter , “Intrabolus Pressure on High‐Resolution Manometry Distinguishes Fibrostenotic and Inflammatory Phenotypes of Eosinophilic Esophagitis,” Diseases of the Esophagus 29, no. 6 (2016): 551–557, 10.1111/dote.12360. 25913144

